# Glomangioma of the Kidney: A Rare Case of Glomus Tumor and Review of the Literature

**DOI:** 10.1155/2017/7423642

**Published:** 2017-06-18

**Authors:** Ammar Almaghrabi, Nizar Almaghrabi, Haneen Al-Maghrabi

**Affiliations:** ^1^Collage of Medicine, Umm Al-Qura University, Makkah, Saudi Arabia; ^2^Department of Pathology, King Faisal Specialist Hospital and Research Center, Jeddah, Saudi Arabia

## Abstract

**Background:**

Glomus tumors are rare mesenchymal tumors originating from glomus bodies in the skin. Glomus tumors of the kidney are rare tumors and only a few cases have been reported in the medical literature. An extensive search revealed a very limited number of primary renal glomus tumors. Although most of these cases were benign in nature, including a case with uncertain diagnosis of malignant potential, two were malignant.

**Case Report:**

We present a unique case of a 57-year-old male patient with an incidentally discovered 2 cm left renal mass. Histopathology examination and immunohistochemical studies confirm the diagnosis of glomangioma (a form of glomus tumor). The patient was followed for one year after partial nephrectomy and showed a benign course without any evidence of local recurrence or metastasis.

**Conclusion:**

To the best of our knowledge, this is the 16th case of primary benign renal glomus tumors. Primary renal glomus tumors are rare and may mimic other mesenchymal renal neoplasms radiologically. Proper investigation (including histopathological analysis and immunohistochemical staining) of kidney tumors is essential to make the diagnosis of glomus tumors, which usually show a benign clinical course following resection.

## 1. Background

Glomus tumors are rare benign mesenchymal neoplasms arising from the neuroarterial receptors called glomus bodies [1]. These are very specialized receptors that comprise an efferent arteriole, anastomotic Sucquet-Hoyer canal, and afferent venule [1]. Any overgrowth and/or hyperplasia in one of these structural parts may result in the formation of glomus tumor. Glomus bodies are normally located in the stratum reticulare of the skin, with greater concentration in the lateral aspects of the digits and the palms [1]. Interestingly, glomus bodies are also found in the precoccygeal soft tissue [2]. These bodies are believed to play a role in thermoregulation [3]. Glomus tumors are rare entities that account for less than 2% of all soft tissue tumors. They are typically localized at the peripheral soft tissues with more tendency to involve the subungual zones of fingers and toes [1, 3]. Visceral organs are rarely prone to develop glomus tumors due to lack or even absence of glomus bodies [4]. An extensive review of the literature revealed only eighteen cases of primary renal glomus tumors. All but three are reported as benign glomus tumors with no evidence of recurrence or metastasis during follow-up [5–17]. These three cases include two cases of malignant glomus tumors [18, 19] and a case of uncertain diagnosis of malignant potential [2]. Our case is the 19th case of glomus tumor of the kidney reported in the world literature and the 16th case of benign primary glomus tumor of the kidney. The 4th edition of the new WHO classification system of the kidney tumors does not include the pericytic tumors and the exceptionally rare glomus tumors [20].

In this study, we discuss the nature of the tumor, challenges in reaching a diagnosis through clinical history and radiological studies alone, and the differential diagnosis to consider. Furthermore, we present a review of all reported cases in the medical literature.

## 2. Case Report

**Figure 1:**
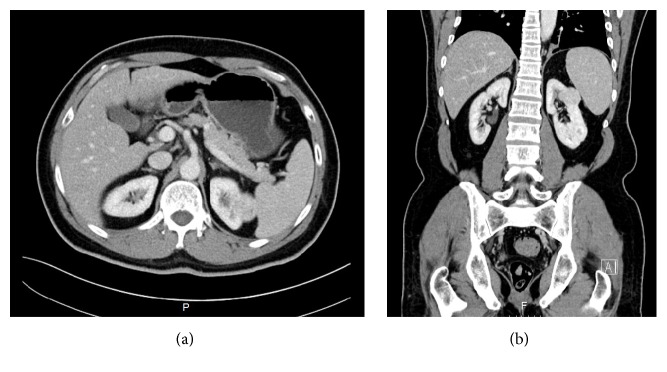
Computed tomography (CT) scan of the abdomen. (a) Axial section and (b) coronal section showing a well-defined heterogeneous lesion measuring 2 × 1.5 cm located at the posterolateral upper pole of the left kidney, most likely arising from kidney cortex.

**Figure 2:**
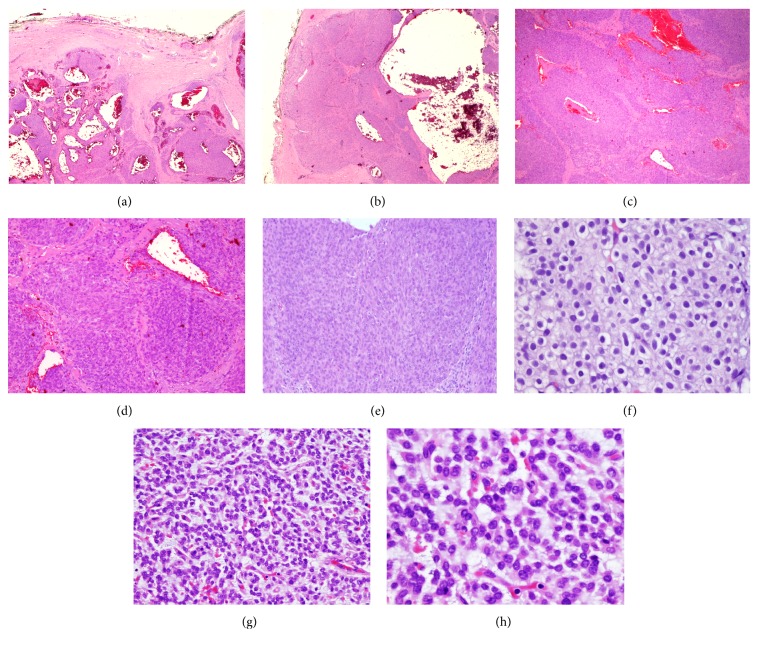
(a) Low-power view of multilobular growth pattern with lobules containing markedly expanded vascular spaces; the lobules are separated by fibrous bands (H&E; ×40). (b) Well-demarcated but uncapsulated tumor demonstrating large gaping vascular spaces surrounded by clusters of glomus cells (H&E; ×100). (c) Tumor cells exhibiting nodular growth pattern, no necrosis seen (H&E; ×100). (d) Sheets and nests of bland cells with oval nuclei with stromal hyalinization (H&E; ×200). (e) Focal areas adjacent to the vascular spaces show solid glomus tumor, consisting of nodules of bland small round to oval monotonous cells with low mitosis (H&E; ×100). (f) Round to ovoid glomus cells with hypercellularity and distinct cell borders, each containing a single centralized, uniform, round, small “punched out” nucleus (H&E; ×400). (g) Glomus tumor forming trabeculae in abundant myxoid areas (H&E; ×200). (h) Small, round, uniform, and with pale eosinophilic to amphophilic cytoplasm (H&E; ×400).

**Figure 3:**
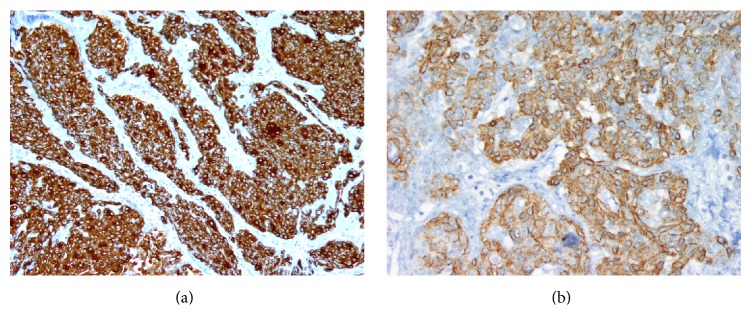
(a) Immunohistochemistry of the tumors cells shows strong and diffuse positivity for alpha smooth muscle actin (*α*-SMA) (H&E; ×200). (b) Nicely uniform pericellular positivity for type IV collagen (H&E; ×200).

A 57-year-old man presented to the hospital with a two-month history of vague “on-and-off” abdominal discomfort. No associated symptoms such radiating pain, weight loss, hematuria, or change in bowel habits were reported by the patient. The patient's medical, surgical, and family history were irrelevant. He also had no history of smoking. Physical examination revealed a soft lax abdomen with unremarkable systemic examination. The results of laboratory investigations, including a complete blood count, blood chemistry, serum urea, and urine analysis, were normal. An abdominal computed tomography (CT) scan showed a well-defined heterogeneous enhancing lesion measuring 2 × 1.5 cm located at the posterolateral upper pole of the left kidney. The lesion was in close proximity to the spleen. There was no evidence of hydronephrosis or kidney stones. The renal vein was patent. These findings suggested renal cell carcinoma (Figure 1). Two weeks later, the patient underwent left partial nephrectomy. The resected specimen was sent for histopathological analysis. Gross examination revealed a well-circumscribed but uncapsulated white-tan soft mass with homogenous cut surface measuring 2 × 1.5 × 1 cm located at the upper pole of the left kidney. The mass abutted but did not invade the renal capsule. No areas of necrosis were seen. No gross abnormality was observed in the rest of the renal parenchyma. Microscopic examination reveals a well-demarcated lesion composed of sheets of cells that were admixed with large, gaping, dilated cavernous-like spaces filled with blood (Figures 2(a) and 2(b)). These cells are monotonous, small, and round to oval, each containing a moderate amount of eosinophilic to amphophilic cytoplasm (Figures 2(e) and 2(f)). No pleomorphism was present. There was no evidence of necrosis (Figure 2(c)) or increased mitotic activity of more than 2/50 high-power field (HPF) (Figure 2(d)). No atypical mitosis was seen. High-power examination showed small nuclei with fine chromatin (Figure 2(f)) and smooth nuclear membrane embedded in a myxoid stroma (Figures 2(g) and 2(h)); other adjacent areas revealed hyalinized stromal reaction (Figure 2(d)). Capsular and lymphovascular invasion were not observed. Tumor cells showed diffuse and strong positivity for smooth muscle actin (*α*-SMA) (Figure 3(a)), vimentin, and pericellular net-like positivity for collagen type IV (Figure 3(b)). The tumor was negative for cytokeratins 7 and 20, RCC antigen, cluster of differentiation (CD10), epithelial membrane antigen (EMA), desmin, CD34, CD117, CD 99, synaptophysin, chromogranin, S100, renin, Melan A, and human melanoma black-45 (HMB-45). Periodic acid-Schiff (PAS) stain failed to reveal any cytoplasmic granules in any of the examined cells. CD31 and CD34 highlighted the vascular spaces and capillary network only. Ki67 index was less than 2%. Altogether, histopathology and immunohistochemical revealed findings consistent with primary glomangioma (glomus tumor) of the kidney. One year after surgery, a follow-up examination revealed that the patient was doing well and no tumor recurrence and/or metastasis was detected.

## 3. Discussion

Glomus bodies are a specialized arteriovenous physiological structure containing a rich nerve supply [17]. Glomus tumors were first described in 1924 by Masson [21]. These tumors are perivascular mesenchymal neoplasms composed of cells closely resembling modified smooth muscle cells of normal glomus bodies [3]. Glomus tumors are found equally in both genders, with a slight female predilection in subungual tumors, and are most common in young adults (20–40 years old) [3]. They are typically located at the distal extremities (particularly nail bed) as small, red-blue nodules, often solitary and painful [3]. Complete surgical excision is the treatment of choice, with excellent prognosis in conventional glomus tumor [3, 9, 10]. One-quarter of glomus tumors are found in the visceral organs, which typically lack glomus bodies [17]. Consequently, an accurate diagnosis can be missed. The exact pathogenesis of glomus tumor in the parenchymal organs is not well understood, since most glomus tumors arise in the soft tissue in association with the normally present glomus bodies. Few reported cases in the literature document primary glomus tumor in the female genital tract [22], gastrointestinal tract [3], bone [23], lung [24], mediastinum [25], larynx [26], trachea [27], oral cavity [28], pancreases [29], liver [30], and sinonasal region [31]. Glomus tumors of the kidney are rare, with limited cases reported in the literature (Table 1). The majority of glomus tumors of the kidney are benign in nature. Only 19 cases including the current one (16 benign glomus tumors, one case of uncertain malignant potential, and two malignant glomus tumors) [2, 5–19] have been reported. Most benign glomus tumors were diagnosed in adults (age range 32–81 years), with a male to female ratio of 2 : 1. Nuwayhid et al. reported a 17-year-old male patient with glomus tumor incidentally diagnosed during a regular follow-up for ulcerative colitis [12]. Most patients with glomus tumors have nonspecific signs and symptoms or are discovered incidentally during regular follow-up. The clinical presentation might include vague abdominal pain, flank pain, lower urinary tract symptoms, and microscopic hematuria. These tumors are mostly located in the renal parenchyma. However, Herawi et al. reported a case of renal glomus tumor located in the renal pelvis causing ureteropelvic junction obstruction and severe hydronephrosis [9]. Onishi et al. reported a case of glomus tumor arising in a congenital hypoplastic kidney that was discovered incidentally [13]. While 99% of glomus tumors occur solitary, typically in the adult population [15], only 10% show multiple presentation in familial generalized multiple glomangiomatosis, mostly in children (e.g., multiple glomus tumor syndrome) which is inherited as an autosomal dominant manner and is known to show incomplete penetrance [32].

Histopathology examination of typical glomus tumors shows a mixed variable proportion of glomus cells, smooth muscle cells, and blood vessels [32]. Depending on the prevalence of round glomus cells, vascular smooth muscle cells, and spindle cells resembling smooth muscle, glomus tumors can be subdivided into solid glomus tumor, glomangioma, and glomangiomyoma, respectively [32]. On the other hand, atypical glomus tumors have been classified into several entities. In 1990, Gould et al. classified atypical glomus tumors as locally infiltrative glomus tumors, glomangiosarcoma emerging from a benign glomus tumor, and glomangiosarcoma arising de novo [33]. In 2001, another classification was suggested by Folpe and colleagues based on a study on 52 cases. Their classification included malignant glomus tumor (glomangiosarcoma), glomus tumor of uncertain malignant potential, symplastic glomus tumor, and glomangiomatosis [34]. Furthermore, Folpe and colleagues suggested the following criteria for malignancy: (1) size > 2 cm and subfascial or deep location; (2) atypical mitotic figures; (3) moderate-to-high nuclear grade and mitotic activity (5 mitoses/50 high-power fields) [34]. The absence of metastasis or local recurrence with low cellular proliferation rate in our case supports the benign nature of the tumor. However, it should be noted that the criteria associated with soft tissue glomus tumor aggression may not be predictive for those in the kidney. Generally, benign glomus tumors are typically solid nests of cells within highly vascularized stroma. These vessels are variable in size ranging from small to large ectatic “staghorn-like” vessels or forming glomuvenous malformation as in glomangioma, similar to our case. The tumor cells are arranged around vessels or can be diffuse and nodular or have a sheet-like appearance in highly cellular tumors. The tumor cells are characteristically small, round, uniform, and with pale eosinophilic to amphophilic cytoplasm. Each contains a single centralized, uniform, round, small “punched out” nucleus. Nuclear atypia and significant mitosis are absent. Occasionally, some cases may show features of oncocytic or epithelioid cytomorphology. The surrounding stroma is myxoid to hyalinized in nature. Low proliferative index with no areas of necrosis is usually seen. Immunohistochemical studies are mostly positive in the tumor cells for SMA, caldesmon, and abundant pericellular type IV collagen. Most of these tumors are negative for various cytokeratins, S100, myoglobin, glial fibrillary acidic protein (GFAP), chromogranin, synaptophysin, EMA, CD20, CD45, CD56, CD57, CD117, Melan A, HMB-45, CD34, CD31, and factor VIII antigen [10]. However, Al-Ahmadie et al. reported a case series of three glomus tumors of the kidney in which one of them showed focal CD34 positivity [10]. Interestingly, one study reported focal tumor cells positivity to S100, which was explained by the mixed component of the tumor as glomus tumor cells, nerve, and vessels [35]. One study shows p53 positivity in the malignant areas stronger and prominent compared with the benign areas [36]. Estrogen and progesterone weak positivity were noted in the case of ovarian glomus tumor [37].

The differential diagnoses include solitary fibrous tumor, myopericytoma, paraganglioma, angiomyolipoma, renal hemangioma, juxtaglomerular cell tumor (JGCT), carcinoid tumor, and lymphoma. Less likely differential diagnoses include Ewing sarcoma/peripheral primitive neuroectodermal tumors, leiomyoma, and renal cell carcinoma. A solitary fibrous tumor will usually have a hemangiopericytoma-like pattern that shows a characteristic spindle or oval cell proliferation arranged in a storiform and fascicular pattern embedded within a hyalinized stroma, which strongly reacts against signal transducer and activator of transcription 6 (STAT6) and CD34 [38]. A myopericytoma is a type of the pericytic neoplasms that grows with a pericytomatous appearance with neoplastic cells arranged in a concentric multilayered fashion surrounding the blood vessels and the dilated branching vascular lumina. One recent study reported a strong expression of CD34 [39] in such a tumor. Paraganglioma is composed histologically of a well-circumscribed mass composed of nested growth pattern of tumor cells known as Zellballen pattern, with a highly vascularized fibrous stroma. Paraganglioma is typically positive for synaptophysin and chromogranin, with S100 positivity in the sustentacular cells in between. Angiomyolipoma (AML), one of the perivascular epithelioid cell tumors (PEComa), was excluded in our case by the lack of melanocytic markers. Renal hemangiomas are considered rare kidney neoplasms [40]. Two types of hemangiomas are documented: the capillary and, more commonly, the cavernous type. Both are composed of variable-sized blood vessels and vascular spaces lined by a single layer of endothelial cells. The underlying stroma may show features of hyalinization with red blood cells extravasation and hemosiderin deposition. SMA might show little positivity in the vessel walls, which should not be confused with the prominent diffuse staining seen in the tumor cells of glomus tumors. Juxtaglomerular cell tumor (JGCT) is a very important differential diagnosis. Most of these patients present in the second or third decades of life, with a slight female predilection [41]. JGCT cause signs and symptoms of hyperreninism, hyperaldosteronism, hypokalemia, and poorly controlled hypertension. Unlike our case, the radiology studies of JGCT show a solid and hypovascular mass. Renal angiography shows, in the majority of JGCT cases, a hypovascular tumor, which helps to rule out renal artery stenosis. Morphologically, JGCT might show similar features with glomus tumors. In addition, it might reveal papillary pattern and well-developed tubules lined by cuboidal cells. Scattered lymphoplasmacytic infiltrates can be seen. Ultrastructural examination of the tumor shows the typical sharply angulated membrane-bound rhomboid crystals and polygonal granules of renin, which act directly with rennin antibodies and PAS, respectively. Unlike JGCT, our case presented with a distinct history with no increased renin level. Moreover, renin and PAS special stains failed to show any reactivity with the tumor cells. Kodet and colleagues [42] reported diffuse CD34 and CD117 positivity in one serial study. Both were negative in our case. Carcinoid tumors stain positively for keratin 18, synaptophysin, and chromogranin, which are all negative in our case. Lymphomas stain positive for CD45 leukocyte-specific markers, CD20, and CD3, all of which stain negative in glomus tumors. The least likely differential diagnosis was Ewing sarcoma/peripheral primitive neuroectodermal tumors, which consist of a sheet of monotonous cells with scant cytoplasm traversed by thin fibrous bands. Perivascular pseudorosettes may be seen. These tumors show diffuse strong membranous staining for CD99 together with the supportive cytogenetics of Ewing sarcoma translocation. Leiomyomas are rare tumors of the kidney, which shows whorled white-tan bulging cut surface. Morphologically, they consist of interlacing bundles of smooth muscle, with a cigar-shaped nucleus. They are not associated with the blood vessels, contrary to glomus tumors. Renal cell carcinoma is a common epithelial tumor, although it is difficult to distinguish it radiologically. Histopathological and immune studies are different; our case does not express any of the epithelial markers (cytokeratin and EMA) besides the distinct morphological features. Overall, our case fits perfectly with the diagnosis of glomangioma.

## 4. Conclusions

We have presented the 16th case of primary renal glomus tumor (in the form of glomangioma). Primary renal glomus tumors are rare and may mimic other mesenchymal renal neoplasms radiologically. Furthermore, histological and immunochemical findings in glomus tumors overlap with those of other kidney tumors and may contribute to an inaccurate diagnosis. Proper investigation (including histopathological analysis and immunohistochemical staining) of incidentally discovered kidney tumors is essential to make the diagnosis of glomus tumors, which show a benign clinical course following resection.

## Figures and Tables

**Table 1:** Summary of the reported cases of glomus tumor in the kidney.

Case number	Author [reference]	Age/sex	Presentation	Site	Size	Treatment	Follow-up (months)
*Benign Glomus Tumor*							

(1)	Our case	57/male	Vague abdominal discomfort	Upper pole of left kidney	2 × 1.5 × 1 cm	Partial nephrectomy	NED (12)

(2)	Lazor et al. (2016) [17]	68/female	Incidental (case of bladder cancer, after BCG therapy)	Left renal cortex	1 cm	Partial nephrectomy	NED (7)

(3)	Novis et al. (2016) [16]	66/male	Incidental (elevated PSA)	Right kidney	5.8 × 5.5 × 4.7 cm	Radical nephrectomy	NA

(4)	Venyo et al. (2012) [15]	32/male	Vague upper abdominal discomfort	Left kidney	4 × 2.3 × 4 cm	Partial nephrectomy	NED (20)

(5)	Sasaki et al. (2011) [14]	62/male	Unexplained weight loss, nausea, and anorexia	Left kidney	1.8 cm	Partial nephrectomy	NED (2)

(6)	Onishi et al. (2010) [13]	36/female	Regular follow-up (elevated proteinuria)	Right congenital hypoplastic kidney	1.7 cm	Retroperitoneoscopic nephrectomy	NED (8)

(7)	Nuwayhid et al. (2010) [12]	17/male	Incidental (during workup for ulcerative colitis)	Upper pole of right kidney	2.1 × 1.4 × 1.9 cm	Wedge resection	NA

(8)	Sugimoto et al. (2010) [11]	41/male	Incidental (regular checkup for leukoderma)	Right kidney	1 cm	Partial nephrectomy	NA

(9)	Al-Ahmadie et al. (2007) [10]	36/male	Abdominal tenderness	Anterior interpolar region of the right kidney	2.3 × 3.2 × 3.3 cm	Partial nephrectomy	NED (62)

(10)	Al-Ahmadie et al. (2007) [10]	81/male	Incidental (history of prostatic adenocarcinoma)	Lower pole of right kidney	4 cm	Total nephrectomy	NED (24)

(11)	Al-Ahmadie et al. (2007) [10]	48/male	Incidental	Midpole of the right kidney	7.3 cm	Total nephrectomy	NED (33)

(12)	Herawi et al. (2005) [9]	53/female	Discomfort in right flank, microscopic hematuria, hydronephrosis	Distal renal pelvis, right kidney	2.5 cm	Radical nephrectomy	NED (6)

(13)	Siddiqui et al. (2005) [8]	55/female	Incidental	Lower pole of her left kidney	2 cm	Partial nephrectomy	NA

(14)	Gravet et al. (2015) [7] (article in French)	60/male	Incidental	Left upper pole	2.5 cm	Partial nephrectomy	NED (8)

(15)	Billard et al. (1990) [6] (article in French)	71/male	Incidental	Right renal capsule	NA	NA	NA

(16)	Schwarz et al. (1957) [5] (article in German)	34/female (pregnant)	Flank pain radiating to the back	Renal parenchyma	NA	NA	NA

*Infiltrating glomus tumor of uncertain malignant potential*							

(1)	Gill and Van Vliet (2010) [2]	46/male	Microscopic hematuria	Anterior aspect of the lower pole of the right kidney	7 × 6.5 × 6.5 cm	Radical nephrectomy	NED (15)

*Malignant glomus tumor*							

(1)	Chen et al. (2016) [19]	46/male	Incidental (history of nasopharyngeal carcinoma)	Upper pole of the right kidney	5 × 4.5 × 4 cm	Radical nephrectomy	NED (6)

(2)	Lamba et al. (2011) [18]	44/male	Lower back pain (metastasis of tumor is the primary presentation)	Posterior side of right kidney with distal metastasis	Multiple metastasis; the size of the primary tumor was unknown	Palliative therapy	Died after 6 months

Total	*19 cases*						
